# A study on the clinical effectiveness of a tiered management model for type 2 diabetes based on a three-level linkage mechanism in the context of residency training: a randomized controlled trial

**DOI:** 10.3389/fendo.2025.1618181

**Published:** 2025-10-21

**Authors:** Silin Wu, Tinglian Liu, Lan Chen, Min Wang, Jieyin Deng, Yang Qin

**Affiliations:** ^1^ Department of General Practice, the General Hospital of Western Theater Command, Chengdu, Sichuan, China; ^2^ Shaheyuan Community Service Center, Chengdu, Sichuan, China

**Keywords:** type 2 diabetes mellitus, standardized residency training, three-tiered collaboration, stratified management, community health services

## Abstract

**Background:**

With the widespread implementation of General Practice Residency Training (GPRT) in China, primary care institutions have enhanced chronic disease management capabilities. However, research on tiered management for type 2 diabetes mellitus (T2DM), particularly systematic exploration based on a three-tier collaborative model (primary-secondary-tertiary institutions), remains limited.

**Objective:**

To evaluate the clinical effectiveness of a three-tiered linkage hierarchical management model for T2DM implemented within the GPRT framework.

**Methods:**

This study enrolled 120 T2DM patients from a community-based GPRT site (followed February-November 2024). Stratified by clinical risk factor, patients were randomly assigned (computer-generated sequence, allocation concealed) to an intervention group (n=60) or control group (n=60). The intervention group received tiered management via an“Internet+”platform(telemedicine)coordinated by multidisciplinary teams (general practice residents, mentors, specialists). The control group received standard community care. Fasting blood glucose (FPG), 2-hour postprandial blood glucose (2h-PBG), glycated hemoglobin (HbA1c), and the Summary of Diabetes Self-Care Activities (SDSCA) scale were assessed at baseline, 3, and 6 months.

**Results:**

Baseline characteristics, FPG, 2h-PBG, and HbA1c were comparable (P > 0.05). Post-intervention, the intervention group exhibited significantly greater reductions in FPG, 2h-PBG, and HbA1c than the control group (P < 0.05). SDSCA scores indicated superior improvements in key domains (diet, exercise, blood glucose monitoring, foot care) for the intervention group (P < 0.05). Medium- and high-risk subgroups within the intervention group achieved significantly better glycemic control than their control counterparts (P < 0.05), with no significant difference observed in the low-risk subgroup (P > 0.05).

**Conclusion:**

The three-tiered linkage management model under GPRT significantly improves glycemic control and self-management in T2DM patients. Integrating multidisciplinary teamwork and digital tools, the model’s structure demonstrates effectiveness within the community setting and exhibits potential for wider implementation in broader healthcare contexts, offering valuable policy implications for optimizing chronic disease management.

**Clinical Trial Registration:**

https://www.chictr.org.cn/index.html, identifier ChiCTR2500100827.

## 1 Introduction

Type 2 Diabetes Mellitus(T2DM) is a globally prevalent chronic metabolic disease. Its rising prevalence and mortality attributed to related complications pose severe challenges to global public health (1). As the country bearing the heaviest burden of T2DM worldwide, China had 140 million people living with diabetes in 2021, among whom approximately 72.83 million (51.7%) remained undiagnosed (2). In terms of disease burden, diabetes accounts for 12.8% of deaths in China and causes a 5.1% loss of health-adjusted life years (3). Consistently, the per-capita economic burden of diabetes would increase from $231 to $414 in China that is estimated during 2020-2030, with an annual growth rate of 6.02% (4). Notably, the prevalence of T2DM is exhibiting a significant upward trend, particularly among younger populations, which is closely linked to rapid socioeconomic development and profound shifts in lifestyle patterns (5). This large patient pool not only imposes a heavy disease burden but also entails substantial economic and psychological pressures. Studies have demonstrated that T2DM-related direct medical costs (e.g., medications, hospitalizations) and indirect costs (e.g., productivity loss, caregiving burden) exert enormous economic strain on China’s healthcare system and patient families (6, 7). Meanwhile, the incurability of T2DM, the need for long-term treatment, and the risk of complications significantly increase the incidence of psychological issues such as anxiety and depression among patients, further reducing their quality of life (8).

Although evidence-based medicine has shown that community-based structured health management and intervention measures can effectively improve blood glucose control and quality of life in patients with T2DM (9), significant regional disparities exist in China’s community health service system regarding resource allocation, service capacity, and management models (10, 11). A critical research gap lies in the lack of a unified, efficient, and sustainable hierarchical diagnosis and treatment system as well as a collaborative management mechanism for diabetes. Existing models generally face the following limitations: (1) Unclear hierarchical division and poor referral efficiency: The division of responsibilities between primary medical institutions and upper-level hospitals is ambiguous, and the two-way referral mechanism operates ineffectively, leading to unordered patient flow (12); (2) Insufficient integration of resources and technologies: There is a lack of effective integration of digital health technologies (e.g., remote monitoring, data-sharing platforms) to support continuous management (13); (3) Weak talent collaboration mechanisms: In particular, there is a lack of systematic research and practical models on how to effectively integrate general practitioners from general hospitals (with both general practice concepts and specialized knowledge), specialists (providing technical support), community family doctors (responsible for daily management and follow-up), and talent resources trained through the standardized residency training (SRT) system for general practitioners.

In recent years, the “Internet+” hospital-community collaborative model has shown potential to improve management efficiency and effectiveness for conditions such as hypertension and obesity (14, 15). However, for patients with T2DM, particularly in terms of hierarchical management based on precise risk assessment and the use of digital technologies, high-quality evidence on effectiveness and generalizability is still limited (16). One area that remains especially underexplored in China is how to integrate residency-trained general practitioners (GPs) from the SRT system into collaborative networks. These GPs, who possess standardized chronic disease management skills, could work alongside community family doctors, hospital-based GPs, and specialists to form a“three-level collaborative”mechanism.

In this mechanism, community family doctors and residency-trained physicians would provide the foundation, handling routine follow-up and initial interventions. General hospital GPs would offer technical guidance, manage moderately complex cases, and coordinate referrals. Specialists would focus on refractory or severe cases, developing individualized treatment plans. Such an approach could enable hierarchical, graded, and continuous management of T2DM. Yet, research on this model is still scarce domestically.

Therefore, this study closely builds upon the core elements of Wagner’s (17) Chronic Care Model (CCM) and, through targeted extensions, proposes a “dual empowerment (education-clinical)” three-level collaborative stratified management model. The core innovations of this model are outlined as follows:“ (1) Systematically integrating community family doctors, residency-trained physicians, general practitioners from general hospitals, and specialist teams, clarifying roles and responsibilities at each level to construct a collaborative network; (2) Utilizing internet platforms to achieve real-time sharing of patient information, remote consultation, and online education, supporting hierarchical management decisions (e.g., formulating differentiated follow-up frequencies and intervention intensities based on clinical risk factor.); (3) Fully leveraging the core chronic disease management capabilities of residency-trained physicians enhanced through standardized training, positioning them as a strong supplement to primary management forces and a talent reserve for the future”.

This study aims to evaluate the impact of this model on blood glucose control, self-management behaviors, and capabilities in T2DM patients, with the goal of exploring a new, scientifically effective, and promotable diabetes management pathway that aligns with China’s national conditions. It intends to provide empirical evidence and practical references for optimizing the primary chronic disease management system.

## 2 Subjects and methods

### 2.1 Study subjects

#### 2.1.1 Recruitment and screening of subjects

This study was designed as a randomized controlled trial. This trial was registered on the WHO international clinical trial registration platform on April 15th, 2025 through China Clinical Trial Registration Center (ChiCTR250010082) and has not been published on other platforms. Based on literature reports (18), glycosylated hemoglobin (HbA1c) was selected as the primary efficacy indicator, and the sample size was calculated using the formula for comparing means between two independent samples.


$$\text{n}=2{\left((\text{Zα}+\text{Zβ})\text{σ}/\text{δ}\right)}^{2}$$

In the formula, the significance level was set at α = 0.05 (two-tailed test), the test power at 1−β= 90%, Zα = 1.96, Z_β_= 1.28,σ= 0.74, andδ= 0.5. The calculation showed that a minimum sample size of 46 cases per group was required. To address potential attrition bias and data fluctuations during study implementation, the sample size was further expanded by 30% based on pre-experimental results. Ultimately, 60 patients were enrolled in both the intervention group and the control group, resulting in a total sample size of 120 cases.

From February 1, 2024, to May 31, 2024, eligible T2DM patients were consecutively recruited from Shaheyuan Community Health Service Center—the primary practice base for SRT in general practice at the General Hospital of Western Theater Command, a collaborating institution of the project. The recruitment method was active screening: Researchers initially identified a list of T2DM patients with regular follow-ups at the center through the electronic health record system of the community health service center. Subsequently, co-enrolled general practice master’s students and general practice residency trainees, who had received unified training, actively invited eligible patients to participate in the study via telephone or during outpatient follow-ups in accordance with pre-defined inclusion and exclusion criteria.

➢ A total of 166 patient records meeting the preliminary diagnosis of T2DM were included in the initial screening. After a detailed evaluation based on inclusion and exclusion criteria, 120 eligible patients ultimately signed the informed consent form.

➢ Inclusion criteria:

- (1) Meeting the diagnostic criteria specified in the Guidelines for the Prevention and Treatment of Type 2 Diabetes in China (19), with a HbA1c level of ≥6.5% in the most recent test before enrollment (≤3 months prior to enrollment);
- (2) Aged ≥40 years and <90 years;
- (3) Possessing basic communication and expression abilities to cooperate with completing scales and questionnaires;
- (4) Able to use smartphones and the Internet daily independently or with assistance from family members;
- (5) Voluntarily participating in the study and signing the informed consent form.

➢ Exclusion criteria:

- (1) Presence of severe acute or chronic complications of T2DM;
- (2) Presence of severe comorbidities or end-stage conditions of systemic diseases;
- (3) Other conditions deemed unsuitable for enrollment after evaluation (e.g., pregnant women, patients with cognitive impairment);
- (4) Refusal to sign the informed consent form.

➢ Exclusion criteria during the study: Patients lost to follow-up during the study (unreachable or voluntary withdrawal), or those who failed to complete the key follow-up points at 3 months and 6 months as planned (including HbA1c testing and core scale assessment).

#### 2.1.2 Risk stratification and grouping

After enrollment, patients were stratified according to their HbA1c levels and complication status. The stratification criteria were developed with reference to two guidelines: Guidelines for the Prevention and Treatment of Type 2 Diabetes Mellitus in China (2020 Edition) (20) and Pharmacologic Approaches to Glycemic Treatment: Standards of Care in Diabetes—2023 (21), while also taking into account the management objectives of the present study.

➢ High-risk stratum: HbA1c > 9.0%, or occurrence of ≥2 symptomatic hypoglycemic events (blood glucose <3.9 mmol/L) within the past 6 months, or comorbidity with ≥2 confirmed diabetic microvascular or macrovascular complications (e.g., diabetic retinopathy, diabetic nephropathy [eGFR <60 ml/min/1.73m²or proteinuria], diabetic neuropathy, coronary heart disease, ischemic stroke, peripheral arterial disease);
➢ Moderate-risk stratum: 7.0%<HbA1c ≤ 9.0%, or comorbidity with 1 confirmed diabetic complication;
➢ Low-risk stratum: 6.5%≤HbA1c ≤ 7.0% without significant clinical symptoms of diabetic complications.

Based on the above criteria, among the 120 enrolled patients, 41 were classified as high-risk, 54 as moderate-risk, and 25 as low-risk. Grouping was performed using a stratified randomization method, following the steps below: First, patients were stratified by their risk level (high, moderate, low). Within each stratum, participants were randomly allocated at a 1:1 ratio to either the intervention group (n=60) or the control group (n=60) using a computer-generated random number sequence. To ensure the integrity of randomization, allocation sequence concealment was strictly maintained until the participants were fully enrolled and assigned to their respective intervention groups. Specifically, the computer-generated random number sequence was generated and stored by an independent statistician who was not involved in patient recruitment or enrollment. Enrollment staff only received the group assignment (intervention or control) for each patient from the independent statistician after confirming that the patient met all enrollment criteria and had completed the enrollment process. The intervention group received the internet-based three-level collaborative hierarchical management model intervention, whereas the control group continued with the routine community follow-up management protocol.

### 2.2 Intervention methods

#### 2.2.1 Intervention for the intervention group

Internet-Based Three-Level Collaborative Hierarchical Management Model.

Participants in the intervention group received a 6-month structured intervention using the internet-based three-level collaborative hierarchical management model. Regarding blinding status.

➢ Participants: All participants were explicitly informed of their group assignment (intervention or control) before the trial initiation. This was necessary because the intervention involved active engagement with internet-based management tools (e.g., remote monitoring platforms, personalized guidance), which differed substantially from the routine follow-up provided to the control group, making participant blinding impractical.
➢ Intervention deliverers and carers: Due to the nature of the intervention— which required dedicated staff to deliver internet-based guidance, review real-time data, and adjust management plans—blinding of the intervention deliverers and associated carers was not feasible. These personnel were fully aware of participants’ group assignments to ensure proper implementation of the intervention protocol.

The core elements of the 6-month structured intervention were as follows:

(1) Management Team Establishment and Standardized Training:

➢ Team Composition: Upper-Level Hospital Team: Composed of 2 senior general practice attending physicians (with≥5 years of experience in general practice/endocrinology) and 4 specialist attending physicians from the General Hospital of Western Theater Command (1 each from endocrinology, nephrology, cardiovascular medicine, and nutrition departments, all with associate chief physician or higher titles), providing remote and offline multidisciplinary support.
➢ Community Team: Composed of 2 community general practice attending physicians (attending physician or higher), 4 community nurses (charge nurse or higher, all trained in chronic disease management), and 2 general practice residency trainees who had completed ≥18 months of standardized training from Shaheyuan Community Health Service Center, responsible for the implementation of intervention measures.
➢ Standardized Training and Standard Operating Procedure (SOP): Prior to intervention implementation, all team members (including residency trainees) received 2 days (16 hours) of centralized standardized training. Training content included: detailed explanation of the study intervention protocol, responsibilities and procedures of three-level collaboration, key points of guideline-based hierarchical management of T2DM ( including hypoglycemia identification and management), internet platform operation specifications, effective communication skills, and patient self-management support strategies. Only those who passed the post-training assessment were allowed to participate in the study. The team strictly followed the pre-developed SOP for Three-Level Collaborative Hierarchical Management to perform all tasks, ensuring intervention homogeneity.

(2) Establishment of Electronic Health Records and Risk Stratification: Research assistants established standardized electronic health records for each patient in the project-specific data collection database using a unified template. Record content included: demographic information, detailed medical history (diagnosis time, past complications, comorbidities, medication history), baseline physical examination (height, weight, BMI, blood pressure), laboratory test results (HbA1c, fasting blood glucose, blood lipids, liver and kidney function, etc.), and lifestyle assessment (diet, exercise, smoking, alcohol consumption). Based on this record information and the aforementioned risk stratification criteria (see 2.1.2), the initial risk level (high, moderate, low) of each patient was jointly reviewed and confirmed by community general practice attending physicians and one upper-level hospital general practice attending physician.

(3) Risk-Stratified Differentiated Intervention Protocol Intervention intensity and core responsible teams were dynamically adjusted based on risk levels:

High-risk subgroup (n=22):

➢ Leading team: General practitioners from upper-level hospitals + relevant specialist attending physicians (based on complication status). Community role: Community team (including residency trainees) provided close assistance, responsible for daily monitoring, patient education, and feedback on protocol implementation.
➢ Core intervention: Within 2 weeks of enrollment, the upper-level hospital team led a multidisciplinary team (MDT) online/offline consultation (22) to develop individualized intensive treatment plans (medication, insulin, diet, exercise, monitoring).
➢ Tasks of residency trainees: Under the supervision of community attending physicians, they proactively contacted patients at least once weekly (via phone/WeChat group messages), focusing on monitoring blood glucose fluctuations (especially hypoglycemia), symptom changes, treatment adherence, and psychological status, and promptly reported abnormalities to the upper-level team.
➢ Blood glucose monitoring requirements: At least daily fasting and postprandial (3 meals) fingertip blood glucose monitoring; data were collected and entered into the established database in real-time by project residency trainees.
➢ Team follow-up frequency: MDT team conducted monthly online re-evaluation; community team (including residency trainees) performed structured follow-up every 2 weeks.

Moderate-risk subgroup (n=28):

➢ Leading team: Community family doctors (community general practice attending physicians). Upper-level support: Upper-level hospital general practitioners provided on-demand technical consultation (platform online consultation or appointment-based referral).
➢ Tasks of residency trainees: Assisted family doctors in patient education, follow-up plan implementation, and data collation, with routine follow-up once every 2 weeks.
➢ Core intervention: Individualized management plans were formulated by community family doctors under the guidance of upper-level hospital general practitioners.
➢ Blood glucose monitoring requirements: Monitoring on at least 3 days per week (including 1 rest day), with daily fasting and 1–2 postprandial blood glucose measurements; data were summarized and uploaded to the platform weekly.
➢ Team follow-up frequency: Community team conducted monthly structured follow-up (in-person/video); upper-level team intervened based on alerts or requests from patients/community doctors.

Low-risk subgroup (n=10):

➢ Leading team: Community health team (with nurses and residency trainees as core members, supervised by family doctors).
➢ Core intervention: Emphasis on patient self-management, with the goal of maintaining stability.
➢ Tasks of residency trainees: Proactive contact once monthly to provide supportive consultation, strengthen health education, and address questions. Blood glucose monitoring requirements: 1–2 paired measurements per week (e.g., fasting + 1 postprandial), with data uploaded monthly.
➢ Team follow-up frequency: Community team performed structured follow-up every 2 months (focusing on education and behavior maintenance); patient self-monitoring records were emphasized.

(4) Internet-Supported Continuous Health Education and Interaction Platform: Primarily relying on the project-specific WeChat mini-program and auxiliary WeChat groups.

➢ Content and Frequency: Structured core diabetes knowledge (covering diet, exercise, medication, monitoring, complication prevention, foot care, psychological adjustment, etc.) is pushed through WeChat groups twice a week. The content is uniformly developed and reviewed by the research team based on authoritative guidelines (19, 23).
➢ Team doctors/nurses/residency trainees check patient questions on the platform/group at least once daily on working days and provide professional and standardized responses within 24 hours. Online thematic micro-courses or Q&A live sessions are organized once a month.

(5) Digital Follow-Up, Monitoring, and Dynamic Adjustment Patients are required to upload the following data through the WeChat mini-program: daily/weekly/monthly blood glucose levels (based on risk stratification), weekly exercise records (type, duration, frequency), diet diaries (with photos), medication records, and subjective feelings (e.g., sleep, mood). The community team (especially residency trainees) is responsible for daily monitoring of platform data. The WeChat mini-program is equipped with automatic alerts (e.g., blood glucose exceeding the target range, continuous unrecorded data). When alerts or abnormal/missing data are detected, the responsible team members (corresponding to the risk stratification) must proactively contact the patient within 24 hours to verify the situation, assess the causes, and conduct preliminary handling or escalate the report in accordance with SOP. The intervention plan (medication, monitoring frequency, education focus) is dynamically evaluated and adjusted by the responsible team based on patient-uploaded data, follow-up feedback, and re-examination results such as HbA1c. In principle, a formal evaluation is conducted every 1–3 months (based on risk stratification).

(6) Monitoring of Patient Engagement and Adherence to Internet Platform Usage:

➢ Records and calculations include:

- a) Login frequency of the WeChat mini-program;
- b) Completion rate of key data uploads (calculated as: number of blood glucose records uploaded as required / total number of records that should be uploaded × 100%);
- c) Reading rate of educational materials (proportion of pushed reading materials that are clicked and viewed).

➢ WeChat Group Engagement: Records include:

- a) Group joining rate;
- b) Average message reading rate (proportion of group messages that are read);
- c) Number of active questions raised. Follow-Up Adherence: Records the actual completion rate of scheduled follow-ups (in-person/phone/video).

➢ Comprehensive Evaluation: The above quantitative indicators serve as important references for assessing patients’ overall adherence to the intervention. Patients with low adherence (e.g., failure to upload key data for 2 consecutive weeks without responding to reminders) trigger an additional follow-up process.

(7) Measures to Address Technical Barriers Baseline Assessment and Technical Training:

➢ At enrollment, patients’ (and assisting family members’) ability to operate smart devices is evaluated. For those unfamiliar with operation (especially elderly patients), residency trainees or community nurses provide one-on-one, hands-on initial training (≥30 minutes), covering core functions such as opening the WeChat mini-program, account login, data entry and upload, message checking, and participation in online WeChat group communication.
➢ Continuous Technical Support: A dedicated technical support hotline is set up (staffed by residency trainees familiar with the platform on a rotating basis) to resolve daily operational issues.
➢ Low-Technology Threshold Options: Patients are allowed to report data verbally via phone for entry into the platform by family members or team members; key educational materials are provided in paper format as an alternative; important notifications are simultaneously delivered via SMS or phone calls.
➢ Involvement of Family Members/Caregivers: Primary family members/caregivers are encouraged and trained to assist in using WeChat groups and the WeChat mini-program, especially for elderly patients or those with operational difficulties.

#### 2.2.2 Control group intervention

Routine Community Follow-Up Management Patients in the control group received the current routine T2DM follow-up management protocol at Shaheyuan Community Health Service Center for 6 months. The protocol mainly includes: Follow-Up Executors: Community nurses. Follow-Up Frequency and Form: Once a month, primarily via telephone follow-up, combined with outpatient follow-up or home visits when necessary. Follow-Up Content: Inquiring about patients’ recent self-monitoring of blood glucose and blood pressure (relying on patient self-reporting or records), assessing medication adherence, diet and exercise status, and psychological state. When potential issues are identified (e.g., poor blood glucose control, symptom changes), nurses develop simple care plans (e.g., reminding patients to revisit the clinic, strengthening monitoring) and evaluate the effectiveness during the next follow-up. The internet platform, structured health education pushes, active data monitoring alerts, or three-level collaborative expert support used in this study are not provided. Patients undergo re-examinations such as HbA1c testing as required by routine outpatient services.

### 2.3 Observation indicators

Note: To minimize assessment bias, all outcome assessors were blinded to participants’ intervention group assignments (i.e., unaware of whether a participant received the internet-based management model or routine follow-up). No assessor was involved in participant recruitment, intervention delivery, or data entry related to group allocation.

#### 2.3.1 Blood glucose control indicators

To objectively evaluate the intervention effect, the following blood glucose indicators were measured at three time points (baseline: before intervention initiation; end of Month 3; end of Month 6) by dedicated, trained personnel as specified below:

#### 2.3.1.1 Fasting plasma glucose

➢ Sample Collection Assessors: Two uniformly trained registered nurses (certified in clinical venipuncture by the Shaheyuan Community Health Service Center) who were not involved in the study’s intervention implementation. These nurses confirmed participants’ overnight fast duration (≥8 hours) before sample collection.
➢ Detection Assessors: Three certified clinical laboratory technicians (with 5+ years of experience in biochemical analysis) at the Shaheyuan Community Health Service Center’s Clinical Laboratory. These technicians operated the Mindray BS-480 automatic biochemical analyzer, followed standardized GOD-POD method protocols, and reviewed internal quality control (IQC) results daily to validate data accuracy (24).
➢ Quality Oversight: One clinical laboratory supervisor (with a master’s degree in clinical laboratory science) reviewed 10% of randomly selected FPG test records monthly to ensure compliance with detection standards.

##### 2.3.1.2 2-hour postprandial blood glucose

➢ Sample Collection Assessors: The same two trained registered nurses responsible for FPG sample collection (to ensure consistency in venipuncture technique). These nurses distributed standardized breakfasts (75g available carbohydrates) and timed the 2-hour interval precisely before sample collection.
➢ Detection Assessors: The same team of certified clinical laboratory technicians and supervisor as FPG detection. All 2h-PBG samples were analyzed using the same Mindray BS-480 analyzer and GOD-POD method as FPG, with identical IQC procedures (24).

##### 2.3.1.3 Glycated hemoglobin (HbA1c)

➢ Sample Collection Assessors: The two trained registered nurses at Shaheyuan Community Health Service Center and placed samples in EDTA anticoagulant tubes.
➢ Transport Assessors: Two designated logistics staff (employed by the General Hospital of Western Theater Command) who transported refrigerated samples (4 °C) to the hospital’s Laboratory Medicine Center within 3 hours of collection. These staff only handled sample packaging and transport, with no access to participant group information.
➢ Detection Assessors: Four laboratory technologists at the General Hospital of Western Theater Command’s Laboratory Medicine Center. These technologists operated the Bio-Rad D-10™ HbA1c Analyzer (HPLC method) and followed IFCC-standardized detection systems.
➢ Validity Assessors: One senior laboratory physician at the same center reviewed all HbA1c test reports to confirm alignment with NGSP and IFCC standards, resolving any abnormal results through retesting.

#### 2.3.2 Self-management behavior capability

The Chinese version of the Summary of Diabetes Self-Care Activities Measure (SDSCA) was used to assess patients’ self-management behavior capability. The original scale was developed by Toobert et al (25) and its Chinese version was translated and validated by Li Yanfei et al (26) for applicability in the Chinese type 2 diabetes mellitus population.

##### 2.3.2.1 Scale administration assessors

➢ Primary Assessors: Three trained research assistants who were not involved in intervention delivery. These assistants administered the SDSCA via face-to-face interviews at the community health center, using a standardized script to clarify ambiguous items.

##### 2.3.2.2 Scale scoring assessors

➢ Scoring Team: Two biostatisticians (affiliated with the General Hospital of Western Theater Command’s Clinical Research Center) who were blinded to group assignments. Each biostatistician independently scored 50% of the SDSCA questionnaires, with cross-validation of 20% of overlapping records to resolve scoring discrepancies.
➢ Data Verification: One research coordinator (with experience in clinical trial data management) reviewed all scored SDSCA data for missing values or outliers, consulting the primary assessors only for clarification on ambiguous responses (without disclosing group status).

##### 2.3.2.3 Scale structure

The Chinese version of the SDSCA comprises 10 items across 4 dimensions: dietary management (4 items), exercise management (2 items), blood glucose monitoring (2 items), and foot care (2 items).

##### 2.3.2.4 Scoring method

A Likert-type scoring system was adopted to assess behaviors over the past 7 days. Each item is scored on a 0–7 scale, and the score for each dimension is the average score of its respective items (range: 0–7). The total scale score ranges from 0 to 28.

##### 2.3.2.5 Evaluation criteria

According to the conventions of domestic related studies, the total score was categorized as follows: >23 points indicate“good adherence,”17–23 points indicate “moderate adherence,”and <17 points indicate“poor adherence.”A higher score reflects a better level of patients’ self-management behavior.

##### 2.3.2.6 Reliability and validity

In the study’s baseline data, the Cronbach’s α coefficient for the total SDSCA score was 0.865, indicating good internal consistency.

### 2.4 Quality control

Data were collected using a combination of online questionnaires (via Wenjuanxing platform) and paper-based surveys. Two resident physicians, who underwent standardized and uniform training, were responsible for collecting patient self-management behavior data through on-site surveys conducted at baseline (pre-intervention) and six months post-intervention.

Prior to survey administration, investigators thoroughly explained the study purpose and scale completion procedures to patients to ensure full comprehension. Questionnaires were distributed only after obtaining patients' informed consent. Completed forms were collected immediately on-site after filling.

To ensure data accuracy and completeness, a dual-entry system was implemented: two individuals independently entered the data, followed by verification by a third party.

To minimize potential bias, data analysts were blinded to group allocation during the analysis phase.

Furthermore, baseline characteristics of the experimental and control groups were compared to assess the balance achieved by randomization, thereby ensuring the scientific validity and credibility of the study results.

### 2.5 Statistical methods

Statistical analyses were performed using SPSS 26.0 software. All statistical tests were two-tailed, with the significance level set at α=0.05. Data Processing and Missing Values: A total of 120 subjects who completed baseline assessments were included in the final analysis. Considering attrition during follow-up (103 subjects completed the 6-month follow-up), this study adopted the Intention-To-Treat (ITT) principle as the primary analytical strategy, incorporating data from all randomized subjects (n=120). For endpoint data of lost-to-follow-up subjects (FPG, 2h-PBG, HbA1c, and total SDSCA scores at 3/6 months), multiple imputation (MI) was used for imputation (5 imputed datasets were created, with imputation based on baseline characteristics and available follow-up data), and the analysis results were combined and reported (27). Meanwhile, results of the Per-Protocol Set (PPS) analysis (n=103) were reported as a sensitivity analysis to evaluate the effect of the intervention in compliant subjects.

➢ Regarding the testing for data distribution, the normality of all continuous variables (including FPG, 2h-PBG, HbA1c, SDSCA score, and SDSCA dimension scores) was assessed using the Shapiro-Wilk test. For homogeneity of variance, Levene’s test was applied (to support subsequent use of the independent samples t-test and analysis of variance ANOVA).
➢ For the description and comparison of baseline data, continuous variables with a normal distribution were expressed as mean ± standard deviation (mean ± SD), while non-normally distributed continuous variables were presented as median (interquartile range) [M (P25, P75)]. Categorical variables were reported as frequency (percentage) [n (%)]. When comparing baseline characteristics between the intervention and control groups: the independent samples t-test was used for normally distributed continuous data; the Mann-Whitney U test was applied for non-normally distributed continuous data; and the chi-square test (χ² test) or Fisher’s exact test (when >20% of cells had an expected frequency <5) was employed for categorical data.
➢ For the analysis of primary outcome indicators (blood glucose indicators), repeated measures data analysis was conducted for variables measured at three time points (baseline, 3 months, and 6 months), including FPG, 2h-PBG, and HbA1c. Two-way repeated measures ANOVA was used for this purpose, with the following factors included: group factor (intervention group vs. control group), time factor (baseline, 3 months, 6 months), and group × time interaction. Mauchly’s test was used to assess the sphericity assumption; if sphericity was satisfied (p>0.10), standard ANOVA results were reported, whereas the Greenhouse-Geisser correction was applied if sphericity was violated. If the group × time interaction was statistically significant (p<0.05), further simple effects analysis was performed to examine between-group differences at each time point and within-group changes across time points. If the interaction was not significant (p≥0.05) but the main effect of group or time was significant, the respective main effect (group or time) was reported independently.
➢ For multiple comparison correction, the Bonferroni method was used to adjust for pairwise comparisons in two scenarios: comparisons between multiple levels of the time factor (baseline vs. 3 months vs. 6 months) in repeated measures ANOVA, and pairwise comparisons in simple effects analysis. This correction was implemented to control for inflation of Type I error (28).
➢ Regarding the reporting of effect sizes, statistically significant comparisons were supplemented with effect size metrics to quantify the magnitude of differences: For the independent samples t-test or Mann-Whitney U test, Cohen’s d (for t-test) or r = Z /√N (for Mann-Whitney U test) was reported. For repeated measures ANOVA (including main effects of group and time, and group × time interaction), partial Eta squared (ηp²) was converted to Cohen’s d to reflect the proportion of variance explained by the effect. Cohen’s d values were interpreted as follows: 0.2 (small effect), 0.5 (medium effect), and ≥0.8 (large effect) (29).
➢ For the secondary analysis (SDSCA dimension scores), changes in SDSCA dimension scores (dietary management, exercise management, blood glucose monitoring, and foot care) between baseline and 6 months were analyzed using the paired t-test if the data met normality assumptions; if not, the Wilcoxon signed-rank test was used. Corresponding effect sizes (e.g., Cohen’s d for paired samples) were reported for these analyses.

## 3 Results

### 3.1 Baseline characteristics

A total of 120 patients were initially enrolled in the study, with 103 completing the entire follow-up period. Of the 17 patients who were lost to follow-up, the breakdown by group was as follows:

➢ Intervention group: A total of 9 patients were lost to follow-up. The reasons for loss included work-related time conflicts (n=4), relocation outside the study’s service area (n=2), and voluntary withdrawal from the study (n=3). This resulted in 51 patients (out of the initial 60) completing the study in the intervention group.
➢ Control group: A total of 8 patients were lost to follow-up. The reasons included work-related time conflicts (n=2), voluntary withdrawal (n=4), and the need for further medical treatment for concurrent diseases unrelated to the study (n=2). This resulted in 52 patients (out of the initial 60) completing the study in the control group.

**Figure 1:**
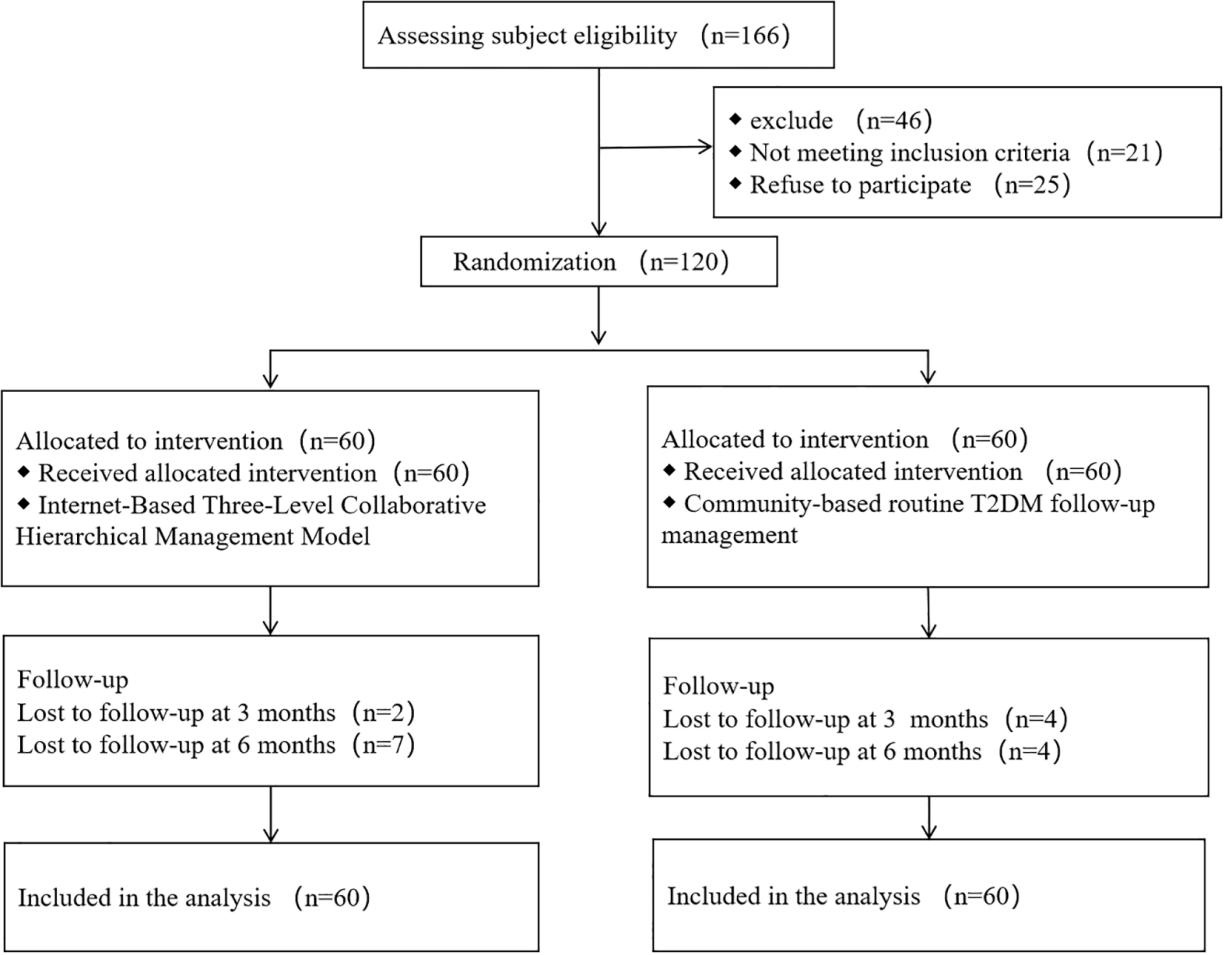
CONSORT flow diagram of the study design.

The flow of participants through enrollment, follow-up, and completion is illustrated in **Figure 1**.

A total of 120 participants were randomized and included in the ITT analysis (**Table 1**). No significant differences were observed between the control and intervention groups in demographic factors (age, sex, BMI, education), clinical profiles (treatment modality, complications, diabetes duration), glycemic parameters (FPG, 2h-PBG, HbA1c), self-management behavior (SDSCA), or risk stratification (all P>0.05). The balanced baseline characteristics confirm successful randomization.

**Table 1:** Baseline demographic and clinical characteristics by group (Intention-to-Treat Population, N=120).

Characteristic	Control group (n=60)	Intervention group (n=60)	Statistics	P-value	Effect size
Demographics					
Age (years), Mean ± SD	63.4 ± 7.0	65.7 ± 8.5	t=-1.627	0.106	d=0.297
Male, n (%)	27 (45.0)	29 (48.3)	χ²=0.033	0.855	φ=0.017
BMI (kg/m²), Mean ± SD	25.0 ± 3.2	25.9 ± 2.1	t=-1.800	0.075	d=0.329
Education Level, n (%)			χ²=1.769	0.622	φ=0.121
≤ Primary school	24 (40.0)	32 (53.3)			
Junior high school	19 (31.7)	16 (26.7)			
High school/vocational	15 (25.0)	11 (18.3)			
≥ College	2 (3.3)	2 (3.3)			
Treatment Modality, n (%)			χ²=1.107	0.775	φ=0.096
Diet only	1 (1.7)	1 (1.7)			
Oral agents	41 (68.3)	46 (76.7)			
Insulin only	3 (5.0)	2 (3.3)			
Insulin + oral agents	15 (25.0)	11 (18.3)			
Diabetes Complications			χ²=3.342	0.068	φ=0.167
Present	37 (61.7)	26 (43.3)			
Absent	23 (38.3)	34 (56.7)			
Diabetes Duration, n (%)			χ²=0.330	0.848	φ=0.052
<5 years	19 (31.7)	17 (28.3)			
5-10 years	19 (31.7)	18 (30.0)			
>10 years	22 (36.7)	25 (41.7)			
Risk Stratification, n (%)			χ²=1.294	0.524	φ=0.104
High-risk	19 (31.7)	22 (36.7)			
Medium-risk	26 (43.3)	28 (46.7)			
Low-risk	15 (25.0)	10 (16.7)			

BMI, Body mass index. Effect sizes: Cohen's d for continuous variables, Cramer's V (φ) for categorical variables;d, 0.2 (Small), 0.5 (Medium), ≥0.8 (Large) [Cohen, 1988].

### 3.2 Comparison of glycemic control outcomes

Significant group × time interactions were observed for all glycemic parameters (FPG: F = 4.199, P = 0.031; 2h-PBG: F = 22.241, P<0.001; HbA1c: F = 22.452, P<0.001), indicating differential treatment effects over time(**Table 2**). Significant group × time interactions (all P<0.05) indicate that glycemic control improved more substantially.

**Table 2:** Comparison of blood glucose control between the two groups at different time points (mean ± SD).

Group	Time	Control group (n = 60)	Intervention group (n = 60)	Mean difference (95% CI)	F-value	P-value	Cohen's d
FPG (mmol/L)	Pre-intervention	8.70±2.79	8.26±2.87	-0.442 (-1.464,-0.581)	F_interaction_=4.199	P_interaction_=0.031	-0.375
	3 Months Post intervention	8.49±1.03	7.36±0.95^ac^	-1.128 (-1.486,-0.771)	F_group_=20.966	P_group_​ < 0.001	-0.844
	6 Months post intervention	8.45±1.34	6.80±0.74^abc^	-1.647 (-2.040,-1.253)	F_time_ = 8.634	P_time_ =0.001	-1.540
2h-PBG (mmol/L)	Pre-intervention	13.03 ±1.68	13.21 ± 1.56	0.187 (-0.399,0.772)	F_interaction_=22.241	P_interaction_<0.001	-0.132
	3 Months Post-intervention	12.53 ± 1.92	11.45 ± 1.45^ac^	-1.078 (-1.693,-0.463)	F_group_=39.696	P_group_ < 0.001	-1.158
	6 Months Post-intervention	12.66 ± 1.23	10.38± 1.00^abc^	-2.285 (-2.690,-1.880)	F_time_=37.268	P_time_ < 0.001	-1.996
HbA1c (%)	Pre-intervention	8.28 ± 1.51	8.47± 1.22	0.193 (-0.302,0.689)	F_interaction_=22.452	P_interaction_​<0.001	-0.127
	3 Months Post intervention	8.08 ± 0.92	7.24± 0.82^ac^	-0.836 (-1.150,-0.522)	F_group_​=17.153	P_group_​ < 0.001	-0.749
	6 Months Post-intervention	7.91 ± 1.03	6.77± 0.65^abc^	-1.137 (-1.448,-0.826)	F_time_=41.311	P_time_ < 0.001	-1.253

a indicates a comparison with the pre-intervention period, P < 0.05; ^b^indicates a comparison with 3 months post-intervention, P < 0.05; ^c^indicates a comparison with the control group, P < 0.05. Interaction effect refers to the phenomenon where, when there are two or more independent variables in a study (e.g., intervention group/control group × baseline/3 months/6 months), the degree of influence of one independent variable (group) on the outcome depends on the level of another independent variable (time). In other words, it describes how the intervention effect changes over time.

over time in the tiered management group compared to the usual care group, with the between-group difference widening progressively over the 6-month period. This accelerating effect aligns with the model's core design principle: intensifying support dynamically based on individual risk levels. *Post-hoc* analyses revealed(**Table 2**).

➢ At 3 months, the intervention group exhibited a significantly lower FPG level (7.36 ± 0.95 mmol/L) compared to the control group (8.49 ± 1.03 mmol/L; d =–0.844, 95% CI:–1.49 to–0.77, P < 0.001). By 6 months, the reduction in FPG in the intervention group became more pronounced (6.80 ± 0.74 mmol/L), showing a substantial clinical effect relative to the control group (8.45 ± 1.34 mmol/L; mean difference =–1.65 mmol/L, d =–1.540, 95% CI:–2.04 to –1.25, P < 0.001).
➢ The intervention group also demonstrated greater improvement in 2h-PBG at both 3 months (11.45 ± 1.45 vs. 12.53 ± 1.92 mmol/L; d = –1.158, 95% CI:–1.69 to–0.46, P < 0.001) and 6 months (10.38 ± 1.00 vs. 12.66 ± 1.23 mmol/L; mean difference = –2.29mmol/L, d = –1.996, 95%CI:–2.69 to–1.88, P < 0.001). Notably, 6-month 2h-PBG in the intervention group approached ADA targets (<10.0 mmol/L).
➢ In the intervention group, HbA1c levels exhibited a progressive reduction over the study period: the mean HbA1c was 8.47 ± 1.22% at baseline, decreased to 7.24 ± 0.82% at the end of the 3rd month (with a mean difference of –0.749 compared to the control group), and further declined to 6.77 ± 0.65% by the end of the 6th month. At the 6-month time point, a clinically meaningful between-group difference was observed in favor of the intervention group. Specifically, the mean difference in HbA1c between the intervention and control groups was –1.14% (95%CI: –1.45 to –0.83), with a standardized mean difference of –1.253 and a statistical significance of P<0.001. Regarding goal attainment, the intervention group achieved a mean reduction in HbA1c of 1.14% (95% CI: –1.45 to –0.83), which exceeded the minimal clinically important difference (MCID) threshold of 0.5% established for T2DM. This finding indicates that the improvement in HbA1c observed in the intervention group is not only statistically significant but also clinically meaningful for patient outcomes.

### 3.3 Comparison of self-management behavior scores

Post-intervention SDSCA scores increased significantly across all domains in the intervention group (P<0.001), with particularly striking improvements in blood glucose monitoring (d=2.33) and exercise adherence (d=1.74)( **Table 3**). The total score surged by 4.97 points—more than double the MCID—demonstrating the model’s capacity to transform self-management behaviors. This behavioral shift likely contributed substantially to the observed glycemic improvements (Section 3.2).

**Table 3:** Comparison of self-management behaviour levels between the control and intervention groups before and after intervention (mean ± SD, points).

Imension	Time point	Control group (n = 60)	Intervention group (n = 60)	*T*-value	*P*-value	95% CI	Cohen's d
Dietary Dimension	Pre-intervention	2.97 ± 0.81	3.10± 0.71	-0.961	0.339	(-0.408,0.181)	0.175
	Post-intervention	3.11 ± 0.69	4.12 ± 0.70^a^	-7.938	<0.001	(1.260,1.534)	1.449
Exercise Dimension	Pre-intervention	2.44 ± 1.48	2.75 ± 1.24	-1.235	0.219	(-0.803,0.186)	0.225
	Post-intervention	2.64 ± 0.66	4.13 ± 0.86^a^	-10.597	<0.001	(1.261,1.739)	1.735
Blood Glucose Monitoring Dimension	Pre-intervention	1.26 ± 0.58	1.28 ± 0.45	-0.129	0.897	(-0.020,0.176)	0.024
	Post-intervention	1.32± 0.54	2.66 ± 0.61^a^	-12.745	<0.001	(1.550,2.433)	2.327
Foot Care Dimension	Pre-intervention	1.39 ± 0.51	1.30 ± 0.45	1.037	0.302	(-0.083,0.267)	0.189
	Post-intervention	1.35± 0.64	2.38 ± 0.94^a^	-7.018	<0.001	(1.123,1.740)	1.281
Chinese Version of SDSCA Total Score	Pre-intervention	8.94 ± 2.73	8.32 ± 2.13	1.391	0.167	(-0.263,1.505)	0.254
	Post-intervention	9.0 ± 1.51	13.29 ± 1.53^a^	-15.431	<0.001	(1.732,2.829)	2.817

a indicates a statistically significant difference compared to pre-intervention (P < 0.05).

### 3.4 Comparison of risk-stratified management effectiveness

**Figure 2:**
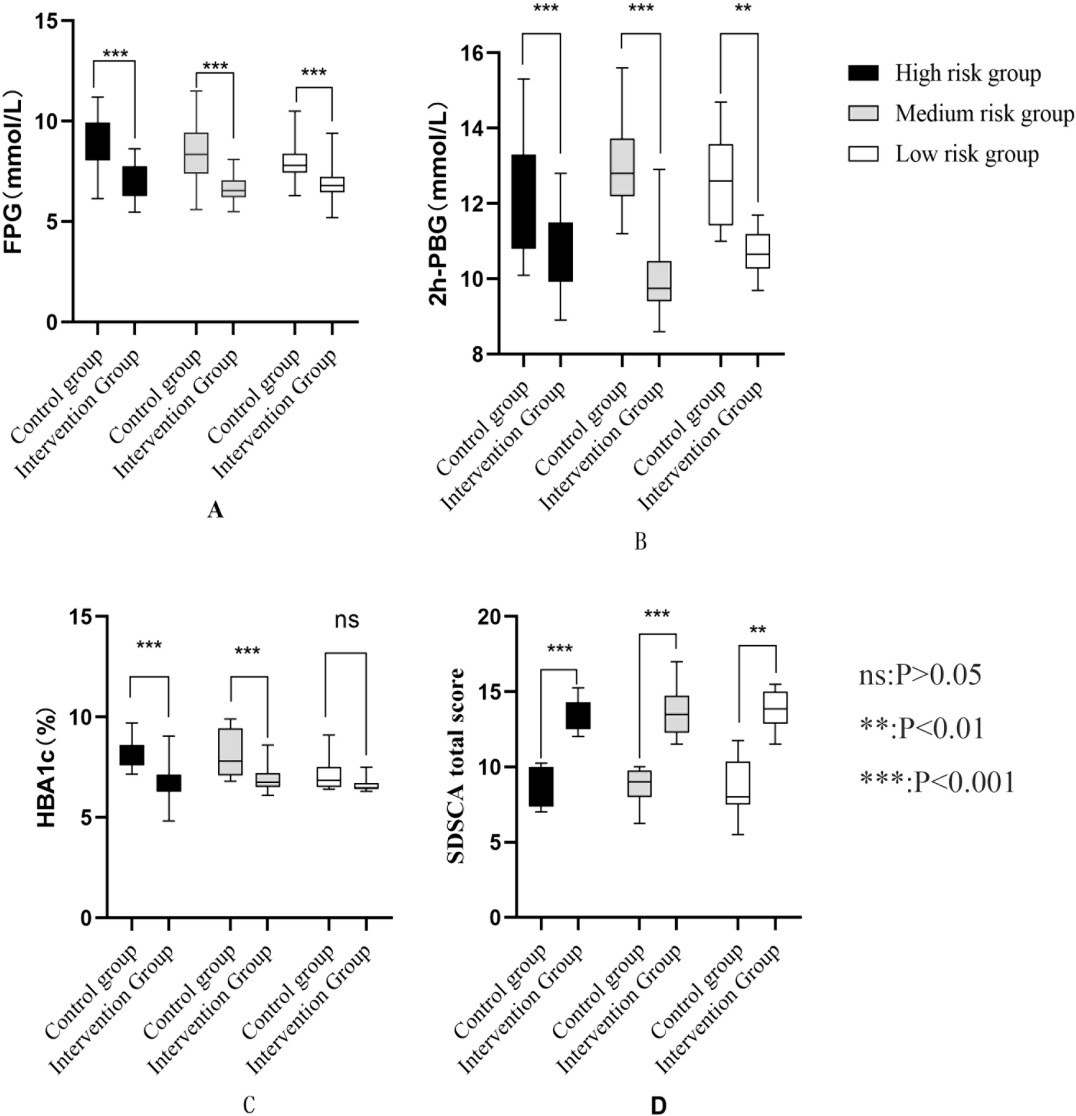
**(A)** Comparison of FPG risk groups after intervention **(B)** Comparison of 2h-PBG in different risk groups after intervention **(C)** Comparison of HBA1c in different risk groups after intervention **(D)** Comparison of self-management behaviors among different risk groups after intervention.

Analysis of key outcomes stratified by risk level (high, moderate, low) revealed significant differences between the control and intervention groups. Detailed results are presented in **Figure 2** and **Appendix 1**.

#### 3.4.1 Fasting plasma glucose

Across all risk strata, the intervention group exhibited significantly lower FPG levels than the control group (all P < 0.001). In the high-risk stratum, the between-group mean difference was −1.87 mmol/L (95% CI: −2.60 to −1.13) with a large effect size (d = −1.611). For the moderate-risk stratum, the mean difference was −1.72 mmol/L (95%CI: −2.35 to −1.10; d = −1.521), while in the low-risk stratum, it was −1.27 mmol/L (95% CI: −1.98 to −0.57; d = −1.524).

#### 3.4.2 2-hour postprandial blood glucose

The intervention group showed significantly lower 2h-PBG levels than the control group across all risk strata (all P < 0.001). Specifically, the between-group mean difference was −1.42 mmol/L (95% CI: −2.20 to −0.64; d = −1.149) in the high-risk stratum, −3.02 mmol/L (95% CI: −3.56 to −2.47; d = −3.026) in the moderate-risk stratum, and −2.03 mmol/L (95% CI: −2.74 to −1.33; d = −2.141) in the low-risk stratum.

#### 3.4.3 Glycated hemoglobin (HbA1c)

In the high-risk and moderate-risk strata, the intervention group had significantly reduced HbA1c levels compared to the control group (both P < 0.001). For the high-risk stratum, the between-group mean difference was −1.57% (95% CI: −2.07 to −1.07; d = −1.982); for the moderate-risk stratum, it was −1.27% (95% CI: −1.75 to −0.79; d = −1.458). By contrast, in the low-risk stratum, the HbA1c reduction in the intervention group (6.62 ± 0.38%) versus the control group (7.03 ± 0.74%) did not reach statistical significance (mean difference: −0.41%, 95% CI: −0.94 to 0.12; P = 0.122) and had a small effect size (d = −0.155).

#### 3.4.4 Summary of diabetes self-care activities score

Across all risk levels, the intervention group demonstrated significantly improved SDSCA scores compared to the control group (all P < 0.001), indicating enhanced self-management behaviors. The mean score increases were 4.31 points (95% CI: 3.34 to 5.27; d = 2.837) for high-risk participants, 4.18 points (95% CI: 3.35 to 5.01; d = 2.764) for moderate-risk participants, and 4.36 points (95% CI: 3.05 to 5.66; d = 2.824) for low-risk participants.

## 4 Discussion

T2DM characterized by its high prevalence, chronicity, and severe complications, poses a substantial burden on patients' quality of life and mental health, representing a major challenge for global chronic disease management systems (30). Robust evidence confirms that glycemic control is paramount in preventing or delaying the onset and progression of diabetic complications (31), with regular glycemic monitoring serving as a cornerstone of effective management (32). Nevertheless, suboptimal adherence to self-monitoring remains a persistent weakness in T2DM self-care, highlighting an urgent need for innovative interventions (33).

### 4.1 Clinical significance of findings

The present study demonstrated that patients in the intervention group achieved significant reductions in FPG, 2h-PBG, and HbA1c from baseline, with outcomes superior to the control group (**Table 2**; **Figure 2**; **Appendix 1**). Notably, the observed HbA1c reductions in the high-risk (-1.57%) and medium-risk (-1.27%) strata (P < 0.001) represent changes of considerable clinical significance. A reduction in HbA1c of ≥0.5% is widely recognized as clinically meaningful, correlating with significant decreases in the risk of microvascular complications over time (34, 35). This magnitude of improvement aligns with findings from comparable tiered management interventions internationally (36–38), reinforcing the value of risk-stratified approaches in T2DM care. The pronounced glycemic improvements, particularly within the high and medium-risk groups (**Figure 2**; **Appendix 1**), strongly suggest that precision stratification enables more effective targeting of interventions. The superior glycemic outcomes in the intervention group, particularly the significant reduction in HbA1c levels, can be mechanistically attributed to the multi-level synergistic system established by the three-level linkage stratified management model. Firstly, structured follow-up and self-monitoring system: Within the three-level linkage model adopted in this study, the healthcare management team implemented standardized predefined follow-up protocols, specifically manifested as: high-risk patients receiving monthly standardized consultations from the multidisciplinary team, and medium-to-low risk patients undergoing hierarchical management by residents in standardized training collaborating with community general practitioners (with follow-up frequency for the low-risk group halved according to standardized procedures). This stratified response strategy effectively overcomes the methodological limitations of the “one-size-fits-all” approach in traditional follow-up through real-time enhancement of treatment adherence monitoring (such as immediate intervention mechanisms for medication non-adherence). Existing studies have confirmed that high-frequency individualized follow-up can significantly improve the magnitude of HbA1c reduction (39). Additionally, combined with standardized guidance protocols for structured self-monitoring of blood glucose (SMBG), patients are ensured to conduct regular and systematic recording of blood glucose changes. A randomized controlled trials have demonstrated that compared with unstructured monitoring, structured SMBG can significantly optimize HbA1c control efficacy and reduce the incidence of hypoglycemic events (40).

Secondly, risk-stratified personalized patient education: The research team implemented customized health education interventions for patients across different risk strata, covering medical nutrition therapy adjustment, individualized exercise prescription formulation, and dynamic assessment systems for medication adherence. A meta-analysis showed that patient-centered educational interventions can significantly reduce HbA1c levels (MD: -0.70%; 95%CI: -0.96% to -0.44%; p=0.001) (41). Finally, real-time physician-patient interaction mechanisms: This study realized dynamic adjustment of treatment regimens and intensive intervention of lifestyle guidance through an internet-supported continuous health education and interaction platform. Empirical research data indicated that irregular follow-up is a strong predictor of poor glycemic control (OR = 4.95, 95%CI: 2.30-11.40, P = 0.001) (42). It can be concluded that sustained physician-patient contact not only significantly improves patient treatment adherence but also enables real-time optimization and precise implementation of intervention measures. In summary, the “three-level collaborative” stratified management model developed in this study under resident training context has effectively improved blood glucose in patients across risk tiers through three core mechanisms—structured follow-up, personalized education, and high-frequency doctor-patient interaction.

### 4.2 Theoretical framework and mechanism of action

The core principle underpinning our model – providing individualized interventions based on clinical risk factor, clinical complexity, and patient needs (43) – resonates strongly with the Chronic Care Model (CCM) (44). Our“tertiary linkage”model operationalizes key CCM elements: health system organization (integrating community health centers, resident physicians, and hospital-based GPs/specialists), decision support (specialist guidance for complex cases), clinical information systems (using the digital platform for data sharing), self-management support (enhanced education and monitoring tools), and community resources (leveraging primary care). The resident physician acted as a crucial “clinical integrator” (45), bridging specialist and community care while leveraging deeper patient understanding to enhance intervention relevance and effectiveness (46). This integrated structure facilitated intensive management for high-risk patients and optimized resource allocation through proportionate intervention intensity across risk strata. Furthermore, this innovative training framework establishes a structured, interdisciplinary learning and clinical practice platform for participating general practice residents, thereby systematically enhancing their interprofessional collaborative competencies. Primarily, within the “three-level linkage” team structure, residents are mandated to engage in collaborative development and implementation of intervention protocols alongside endocrinology specialists, community nursing professionals, registered dietitians, and clinical pharmacists. This immersive clinical environment facilitates the cultivation of sophisticated interdisciplinary communication and collaboration proficiencies within authentic patient care contexts. Empirical evidence has substantiated that structured interprofessional collaborative practice significantly augments residents' comprehension of team role delineation and cooperative dynamics, which subsequently translates into improved patient-centric outcomes (47).

Secondly, the model fosters substantial advancements in clinical decision-making capabilities and informatics proficiency. By leveraging a robust digital infrastructure, it enables precise patient stratification based on multidimensional clinical risk factors, standardized follow-up protocol implementation, and systematic efficacy evaluation. Through iterative engagement with this digital ecosystem, residents develop expertise in utilizing clinical information systems to support evidence-based decision-making processes. Scholarly investigations have demonstrated that computer-based training (CBT) modalities in diabetes management education for residents yield measurable improvements in knowledge retention and clinical decision-making confidence (48). Moreover, the framework promotes reflective clinical practice and longitudinal professional development. Regularly convened multi-tiered case conferences and structured feedback sessions necessitate residents to engage in critical reflection on diagnostic reasoning, therapeutic strategies, communication methodologies, and the integration of humanistic care principles. This cyclical reflective process drives concurrent refinement of clinical judgment acumen and the cultivation of patient-centered care paradigms. Research findings have underscored that cross-institutional clinical collaboration forums provide residents with ecologically valid learning environments, which are pivotal for the development of comprehensive professional competencies in the management of complex chronic conditions (49).

Finally, through the design and implementation of personalized educational interventions tailored to patients across diverse risk stratifications, residents accumulate empirical experience in behavioral motivation theories and health education methodologies. This experiential learning further enhances their capacity for empathetic engagement and effective patient communication, which are integral to holistic patient care delivery.

### 4.3 Enhancement of self-management and theoretical basis

Furthermore, significant improvements in SDSCA scores across all risk levels (**Table 3**; **Figure 2**; **Appendix 1**) were observed in the intervention group, corroborating previous research on self-management enhancement through structured support (50, 51). The integration of“Internet+”technology provided accessible health education channels, bolstering disease knowledge. This aligns with Social Cognitive Theory (SCT) (52), where improved knowledge (knowledge expectations) and accessible tools (environmental factors) enhance self-efficacy, promoting behavioral change (e.g., improved foot care, monitoring, diet, exercise). Consistent with SCT and empirical evidence (53), enhanced knowledge empowers patients towards better glycemic control and improved quality of life. Crucially, self-management capability is a well-established predictor of improved health status, disease control, and quality of life in T2DM (54). Our model’s timely feedback mechanisms, enabled by the digital platform, further reinforced self-monitoring capabilities and motivated adherence, acting as positive performance feedback within the SCT framework (55).

### 4.4 Addressing implementation challenges

While the digital platform enhanced efficiency and communication, potential implementation challenges warrant consideration. Digital divide issues, particularly affecting elderly or socioeconomically disadvantaged populations could limit access and require supplementary low-tech strategies (e.g., phone calls, in-person visits) (56). Workforce constraints, especially in resource-limited primary care settings, necessitate clear protocols, task-shifting where appropriate, and adequate training to ensure sustainable delivery without overburdening staff (57). Ensuring long-term engagement remains a challenge in chronic disease management; strategies like regular motivational support, personalized goal setting, and adapting interventions based on patient feedback are crucial for maintaining participation (58). Proactive mitigation of these challenges is essential for successful real-world scaling.

### 4.5 Scalability, adaptation, and low-risk management

The model demonstrates significant potential for scalability and integration within broader national health systems, particularly those emphasizing primary care and chronic disease management. Its core principles (risk-stratification, multidisciplinary linkage, task-sharing, digital enablement) are adaptable beyond China. The digital component could utilize various existing platforms (e.g., WhatsApp, bespoke apps, EHR-integrated portals) depending on local infrastructure and user preferences (59). Adaptation would require tailoring to specific healthcare structures, workforce capabilities, and cultural contexts. Regarding low-risk patients, while their HbA1c improvement was not statistically significant (P = 0.122, **Figure 2** and **Appendix 1**), likely due to near-target baseline levels and limited intervention headroom, proactive management remains vital. For this group, the focus should shift towards evidence-based prevention and health maintenance (60): reinforcing healthy lifestyle behaviors (diet, physical activity), providing structured education on early warning signs of progression, ensuring annual comprehensive screenings (including cardiovascular risk factors), and fostering long-term adherence to basic self-monitoring. This preventative approach aims to sustain low-risk status, minimize future risk escalation, and prevent complications (61), thereby optimizing resource use by concentrating intensive management on higher-risk individuals while still providing essential support to all.

## 5 Limitations

Although this study verified the effectiveness of the three-level collaborative hierarchical management model under the context of standardized residency training, the following limitations should be objectively stated: First, regarding follow-up completeness and analytical methods: A total of 120 patients were enrolled in this study, with 103 patients completing the 6-month follow-up (attrition rate: 14.2%). The main reasons for attrition included patient relocation leading to loss to follow-up and insufficient adherence to the follow-up process. This study adopted Intention-to-Treat (ITT) analysis, with missing data handled by multiple imputation. While this approach reduced the impact of data missing on the results, missing data in high-risk attrition subgroups (e.g., elderly patients or those with low educational levels) may still introduce bias. Second, potential biases and technical limitations cannot be ignored. In terms of selection bias, all participants in this study were recruited from a single community health service center in Chengdu. The relatively limited sample size and the specificity of the regional population may fail to represent the characteristics of patients in primary care settings or remote areas, potentially restricting the external validity (generalizability) of the results. For measurement bias, self-management behavior scores relied on patients’ subjective reports, which may be subject to recall bias. Regarding technical limitations, approximately 10% of elderly patients failed to fully participate in digital follow-up due to difficulties in using“Internet +” monitoring tools, resulting in lower completeness of data collection compared to younger patients, which may have underestimated the intervention effect. Third, significant challenges exist in the external validity of the implementation scenario. The effective operation of this model depends on close collaboration among “community - standardized residency trainees - general hospitals”. However, in regions with weak medical infrastructure, issues such as insufficient training of primary care physicians, inadequate coverage of specialist resources, and lagging development of digital platforms may arise. These problems could reduce the precision and continuity of hierarchical interventions, affecting the feasibility of replicating the model. Fourth, the 6-month endpoint is consistent with the guidelines of the American Diabetes Association for evaluating glycemic interventions, and a statistically significant reduction in HbA1c was achieved. However, this time frame cannot fully assess the impact of the intervention on long-term diabetic complications (e.g., diabetic nephropathy, peripheral neuropathy) nor verify the long-term sustainability of glycemic control effects. Future studies need to extend the follow-up period to more than 2 years to supplement long-term data. Finally, insufficient control of confounding factors may affect the interpretation of results. This study did not systematically collect and include variables such as medication adherence, daily dietary structure, weekly exercise duration, and levels of psychosocial support, which may independently influence glycemic control outcomes. Although baseline data were balanced between groups, the failure to adjust for the aforementioned variables in the multivariable model may lead to unrecognized confounding biases.

## 6 Conclusion

This study confirms that the residency-based three-level collaborative stratified management model significantly improves glycemic control and self-management abilities in patients with T2DM. Specifically, after 6 months of intervention, the intervention group achieved a significant 1.70% reduction in HbA1c from baseline (from 8.47% to 6.77%), with FPG) and 2h-PBG decreasing by 1.46 mmol/L and 2.83 mmol/L respectively compared to the control group (**Table 2**). Meanwhile, the intervention group showed significantly higher scores in self-management behaviors (including foot care, blood glucose monitoring, exercise, and diet management), indicating the model's dual effectiveness in improving clinical indicators and patient behaviors.

The core value of this model lies in realizing precise stratified intervention and dynamic management of T2DM patients through integrating resources from community health centers, residency physicians, general practitioners, and specialists in general hospitals. The in-depth participation of residency physicians not only strengthens the continuity of inter-institutional collaboration but also provides them with a practical training platform for chronic disease management, contributing to enhancing general practice capabilities and interdisciplinary collaboration awareness. This provides empirical evidence for integrating residency training systems with chronic disease management. At the policy level, the model offers a referable framework for optimizing primary care policies and upgrading national residency programs. Its resource integration mechanism and stratified intervention strategy can support incorporating chronic disease management capabilities into core assessment indicators of general practice residency training, promoting the formation of a “training-practice-quality control”closed-loop system.

Several limitations should be objectively noted: firstly, the single-center design may limit the generalizability of results, as differences in regional medical resource allocation may affect model adaptability; secondly, the 6-month follow-up period is insufficient to evaluate the model’s impact on long-term diabetic complications (e.g., nephropathy, neuropathy); finally, subgroup analysis of patients with digital tool usage barriers was not conducted, which may underestimate the need for non-digital interventions.

Future research can advance in three directions: first, conducting multi-center, large-sample long-term trials to focus on evaluating the model’s impact on diabetic complications and long-term prognosis; second, verifying the model's adaptability in different healthcare systems (e.g., areas with weak primary care resources, private medical institutions) to optimize resource allocation schemes; third, exploring artificial intelligence-integrated dynamic risk prediction models to further improve the precision of stratified interventions, providing evidence for the iterative upgrading of T2DM management models.

## Data Availability

The raw data supporting the conclusions of this article will be made available by the authors, without undue reservation.

## Appendix 1

**Table:** Comparison of glycemic control and self-care behavior outcomes between control and intervention groups stratified by risk level.

Outcome	Risk stratum	Control group (n=60)	Intervention group (n=60)	Mean difference (95% CI)	*P*-value	Effect size
FPG (mmol/L)	High-risk	8.86 ± 1.40	6.99 ± 0.90	-1.866(-2.600,-1.132)	<0.001	d=-1.611
	Medium-risk	8.38 ± 1.42	6.66 ± 0.69	-1.720(-2.345,-1.095)	<0.001	d=-1.521
	Low-risk	8.04 ± 1.02	6.77 ± 0.40	-1.272(-1.977,-0.567)	<0.001	d=-1.524
2h-PBG (mmol/L)	High-risk	12.22 ± 1.39	10.80 ± 1.08	-1.420(-2.204,-0.637)	<0.001	d=-1.149
	Medium-risk	12.96 ± 1.11	9.94 ± 0.88	-3.018(-3.564,-2.473)	<0.001	d=-3.026
	Low-risk	12.71 ± 1.11	10.68 ± 0.58	-2.033(-2.737,-1.330)	<0.001	d=-2.141
HbA1c (%)	High-risk	8.28 ± 0.73	6.71 ± 0.84	-1.569(-2.071,-1.068)	<0.001	d=-1.982
	Medium-risk	8.13 ± 1.09	6.86 ± 0.54	-1.270(-1.751,-0.789)	<0.001	d=-1.458
	Low-risk	7.03 ± 0.74	6.62 ± 0.38	-0.408(-0.935,0.118)	0.122	d=-0.155
SDSCA Score	High-risk	8.93 ± 1.17	13.24 ± 1.84	4.308(3.343,5.272)	<0.001	d=2.837
	Medium-risk	9.21 ± 1.69	13.39 ± 1.33	4.180(3.354,5.007),	<0.001	d=2.764
	Low-risk	8.73 ± 1.62	13.09 ± 1.42	4.355(3.052,5.657)	<0.001	d=2.824
